# Piezoelectric scaffolds for bone regeneration: a systematic review of preclinical studies

**DOI:** 10.3389/fbioe.2026.1848820

**Published:** 2026-06-30

**Authors:** Marco Minelli, Simone Micalizzi, Vincenzo Longobardi, Elizaveta Kon, Paolo Oliva, Federico Della Rocca

**Affiliations:** 1 Department of Biomedical Sciences, Humanitas University, Rozzano, Italy; 2 IRCCS Humanitas Research Hospital, Rozzano, Italy

**Keywords:** bone, regeneration, osteogenesis, piezoelectric, scaffold, tissue engineering, preclinical studies, animal models

## Abstract

**Introduction:**

Critical-sized bone defects remain a major challenge and often require reconstruction grafts. Conventional scaffolds don’t reproduce the dynamic electromechanical behavior of native bone. Piezoelectric scaffolds have emerged as a promising strategy to overcome these limitations by converting mechanical stimuli into bioactive electrical signals capable of enhancing osteogenesis.

**Methods:**

A systematic review was conducted in accordance with the PRISMA 2020 guidelines and registered in PROSPERO (ID: 1359740). A comprehensive search of PubMed/MEDLINE, Scopus, Embase, and Cochrane Central Register of Controlled Trials was performed. *In vivo* animal and clinical studies evaluating piezoelectric scaffolds for bone defect repair were included. Data extraction and risk of bias assessment were performed independently by two reviewers. Outcomes of interest included micro-computed tomography (micro-CT), histological findings, and biomechanical properties.

**Results:**

Ten preclinical *in vivo* studies (2020–2025) were included. No eligible human clinical studies were identified. The included studies involved mainly rodent models, with some studies in rabbits and sheep. Most studies investigated critical-sized calvarial defects, while others evaluated load-bearing models. Piezoelectric scaffolds—primarily based on PVDF, PVDF-TrFE, and BaTiO_3_ composites—consistently demonstrated improved bone regeneration compared with conventional scaffolds. Micro-CT analysis showed higher bone volume fraction (BV/TV) and bone mineral density (BMD) across studies, with BV/TV values reaching approximately 35%–36% at 8 weeks in several models. Histological analyses confirmed enhanced new bone formation, improved tissue organization, and better scaffold–bone integration, often with reduced fibrous tissue formation. Additive manufacturing techniques enabled precise control of scaffold architecture and were widely used, while external mechanical stimulation further enhanced osteogenic outcomes in some studies.

**Conclusion:**

Piezoelectric scaffolds represent a promising approach for bone regeneration, demonstrating superior regenerative performance compared with conventional scaffolds in preclinical models. Their ability to generate bioactive electrical signals, combined with advanced fabrication techniques, supports their potential for clinical translation. However, current evidence is limited to preclinical studies with heterogeneous methodologies. Further standardized studies and clinical investigations are needed to confirm their safety, efficacy, and long-term performance.

## Introduction

Critical-sized bone defects remain a major clinical challenge and often require reconstruction using bone grafts, metal implants, or bioengineered scaffolds (Mayfield et al., 2022; Vidal et al., 2020). Conventional scaffolds composed of polymers, ceramics, metals, or their composites can provide mechanical support and porosity for cell infiltration; however, they largely function as passive structures and fail to replicate the dynamic responsiveness of native bone tissue, which continuously adapts to mechanical, electrical, and biochemical stimuli during the remodeling process (Pereira et al., 2020; Farjaminejad and Dingle, 2025; Roseti et al., 2017). To overcome these limitations, increasing attention has been directed toward smart biomaterials capable of responding to environmental stimuli. These materials may incorporate electroactive, magneto-responsive, thermo-responsive, or pH-responsive elements that enable scaffolds to interact with the biological environment and influence cellular behavior (Xing et al., 2023; Zaszczyńska et al., 2024). Among these strategies, piezoelectric scaffolds have emerged as a particularly promising approach for bone tissue engineering.

Piezoelectric materials generate electrical potentials in response to mechanical deformation. Since bone itself exhibits intrinsic piezoelectric properties, this mechanism is highly relevant for mimicking physiological bone remodeling processes (Zhu et al., 2023). Under mechanical loading, these materials convert mechanical forces into electrical signals that can stimulate osteogenic differentiation and enhance mineralization (Zhu et al., 2023; Omidian and Mfoafo, 2024). Additional developments include stimuli-responsive hydrogels and shape-memory or 4D-printed scaffolds designed to adapt their behavior during the healing process (Ya et al., 2022; Tandon et al., 2018). The rapid expansion of additive manufacturing (AM) technologies has further accelerated the development of such scaffolds by enabling precise control over architecture and material distribution (Gharehdaghi et al., 2024; Xue et al., 2024). Various piezoelectric biomaterials have been investigated for bone regeneration, including poly (vinylidene fluoride) (PVDF), poly (vinylidene fluoride–trifluoroethylene) (PVDF–TrFE), polarized poly (L-lactic acid) (PLLA), polycaprolactone (PCL) and ferroelectric ceramics such as barium titanate (BaTiO_3_) and zinc oxide (ZnO) (Omidian and Mfoafo, 2024). These materials can be processed using a range of additive manufacturing techniques, such as fused deposition modeling (FDM), direct ink writing (DIW), stereolithography/digital light processing (SLA/DLP), selective laser sintering (SLS), and metal-based selective laser melting or electron beam melting (SLM/EBM), which enable precise control over scaffold architecture and spatial distribution of the piezoelectric phase (Pazhamannil and Alkhedher, 2024; Chowdhury et al., 2025). Despite these progresses, evidence remains fragmented across different materials and fabrication approaches. This makes it difficult to identify consistent regenerative trends or clarify which design features are associated with improved outcomes *in vivo*. To our knowledge, no recent systematic review has specifically focused on the *in vivo* performance of piezoelectric scaffolds for bone regeneration while also examining the contribution of additive manufacturing to scaffold architecture and function. The aim of this systematic review was to synthesize the current *in vivo* evidence on the performance of piezoelectric scaffolds for bone regeneration.

Accordingly, this review addresses the following research questions:

RQ1: What types of piezoelectric scaffolds have been evaluated *in vivo* in animal or human models of bone defect repair?
RQ2: How does the regenerative performance of piezoelectric scaffolds compare with that of conventional, non-smart scaffolds?
RQ3: What role does additive manufacturing play in enhancing the structure and function of smart scaffolds?

## Materials and methods

This systematic review was conducted in accordance with the PRISMA 2020 guidelines, and the study protocol was prepared for registration in the PROSPERO database (registration ID: 1359740). The objective of the review was to synthesize available preclinical and clinical evidence on the effectiveness of smart bone scaffolds for bone regeneration.

### Eligibility criteria

Studies were eligible for inclusion if they were randomized or non-randomized clinical investigations or controlled *in vivo* animal studies evaluating bone defect repair using piezoelectric scaffolds. Only full-text articles published in peer-reviewed journals were considered. Studies were included when they evaluated the implantation of a piezoelectric scaffold in an animal or human bone defect model and reported at least one quantitative outcome related to bone regeneration.

For the purpose of this review, piezoelectric scaffolds were defined as three-dimensional biomaterial constructs capable of generating electrical signals in response to mechanical deformation. Eligible interventions included scaffolds composed of or incorporating piezoelectric polymers. Composite systems combining piezoelectric materials with polymers, ceramics, or metallic scaffolds were also considered eligible. Studies were included regardless of fabrication technique, including scaffolds manufactured using additive manufacturing (AM) technologies. Eligible comparator groups included conventional non-piezoelectric scaffolds, empty defect controls, or standard surgical treatments.

Studies were excluded if they were in vitro-only investigations, *ex vivo* experiments, conference abstracts without full text, editorials, letters, narrative reviews, or systematic reviews. Studies were also excluded if they focused solely on material characterization without *in vivo* evaluation, drug delivery systems lacking a structural scaffold function, or soft tissue regeneration unrelated to bone repair.

### Outcomes of interest

The outcomes of interest were quantitative measures of bone regeneration assessed using micro-computed tomography (micro-CT) or histomorphometric analysis, as well as mechanical testing of regenerated bone or implant fixation.

### Information sources and search strategy

A comprehensive literature search was conducted across PubMed/MEDLINE, Scopus, Embase, and the Cochrane Central Register of Controlled Trials. Searches were not restricted by language and included studies published from 2004 to the present, ensuring coverage of the major developments in piezoelectric biomaterials and bone tissue engineering. For PubMed, the following optimized search string was used to identify relevant studies:

[(piezoelectric OR “piezoelectric scaffold” OR PVDF OR “PVDF-TrFE” OR PLLA OR PCL OR “barium titanate” OR BaTiO3 OR ZnO) AND (scaffold OR “bone scaffold” OR “tissue engineered scaffold”) AND (bone OR “bone regeneration” OR “bone defect” OR “critical sized defect”) AND (“*in vivo*” OR animal OR rat OR mouse OR rabbit OR sheep OR goat)].

In addition, the reference lists of all included articles and relevant reviews were manually screened to identify further eligible studies.

### Study selection

Study selection was conducted in two stages. First, two independent reviewers (M.M. and S.M.) screened titles and abstracts to identify potentially relevant studies. Subsequently, full-text articles were retrieved and assessed independently according to the predefined eligibility criteria. Disagreements were resolved through discussion, and when consensus could not be reached, a third reviewer (P.O.) was consulted. The study selection process was documented using a PRISMA 2020 flow diagram.

### Data extraction

Data extraction was performed independently by two reviewers (M.M. and S.M.) using a standardized extraction form. For each included study, information was collected on study characteristics, the animal or human model used, and the location and size of the bone defect. Additional data included scaffold composition, the type of piezoelectric material employed, and the fabrication method, including additive manufacturing techniques when applicable. Details regarding structural and mechanical properties, surface modifications, and the incorporation of bioactive agents were also recorded. All reported outcomes related to bone regeneration were extracted. Furthermore, information on adverse events, complications, funding sources, and potential conflicts of interest was documented. Any discrepancies between reviewers during the extraction process were resolved through discussion and consensus.

### Risk of bias assessment

Risk of bias was evaluated independently by two reviewers (M.M. and V.L.) using tools appropriate for the study design. Animal studies were assessed using the SYRCLE Risk of Bias tool, randomized clinical trials using the Cochrane RoB 2 tool, and non-randomized clinical studies using the ROBINS-I tool. Disagreements were resolved through discussion.

### Data synthesis

Data synthesis followed a predefined analytical framework. When three or more studies reported sufficiently homogeneous outcomes, a random-effects meta-analysis was planned. Continuous variables were summarized using standardized mean differences (SMD) with 95% confidence intervals. Statistical heterogeneity was assessed using the I^2^ and τ^2^ statistics. Prespecified subgroup analyses included type of piezoelectric material, scaffold fabrication method (including additive manufacturing), animal species, and follow-up duration. Sensitivity analyses were planned to assess the robustness of the results. However, quantitative meta-analysis was ultimately not feasible because of substantial heterogeneity across studies in terms of scaffold composition, piezoelectric materials, animal models, defect sites, outcome reporting, and follow-up duration. Therefore, the findings were synthesized using a structured narrative approach.

## Results

### Study selection

**FIGURE 1:**
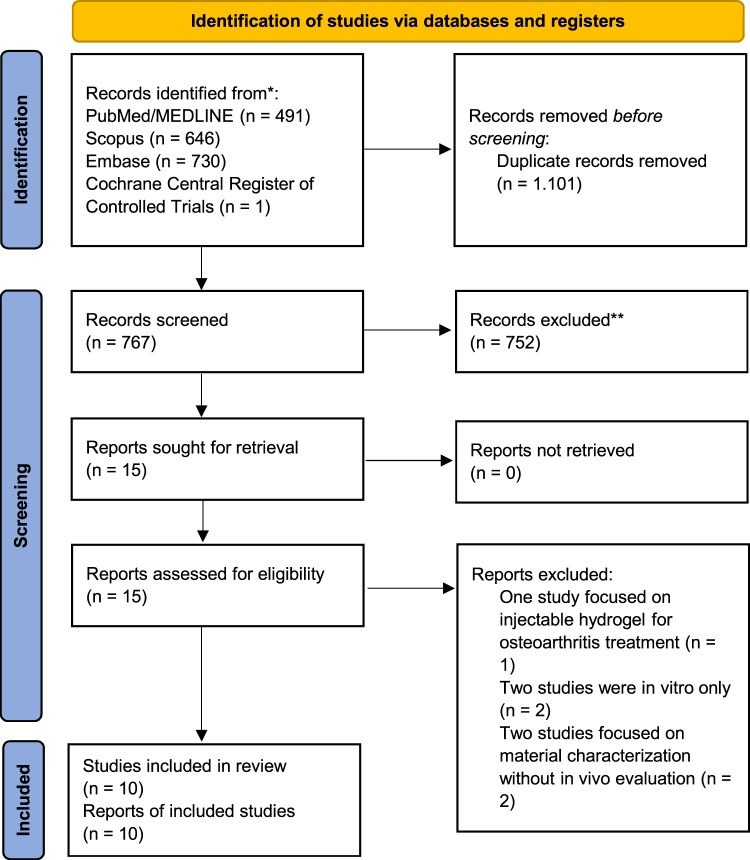
Flowchart of the selection process according to the PRISMA 2020 (Preferred Reporting Items for Systematic Reviews and Meta-Analyses) guidelines.

The database search identified 1868 records, of which 767 remained after removal of duplicates. Following title and abstract screening, 15 articles were assessed for full-text eligibility, and 10 studies met the predefined inclusion criteria and were included in the qualitative synthesis (Wu H. et al., 2023; Wu P. et al., 2023; Zhao et al., 2024; Zheng et al., 2023; Joo et al., 2024; Liu et al., 2020; Li et al., 2025; Fan et al., 2020; Wang et al., 2022; Yong et al., 2025). Although the search strategy considered both preclinical and clinical investigations, no eligible human clinical studies were identified. The study selection process is illustrated in the PRISMA 2020 flow diagram (Figure 1).

### Study characteristics

The characteristics of the included studies are summarized in Table 1. All studies were preclinical *in vivo* experimental investigations, corresponding to Level V evidence. The studies were published between 2020 and 2025 and investigated smart bone scaffolds with electroactive or piezoelectric functionality for the treatment of critical-sized bone defects. Most experiments were conducted in rodent models (Wu P. et al., 2023; Zhao et al., 2024; Zheng et al., 2023; Joo et al., 2024; Li et al., 2025; Wang et al., 2022; Yong et al., 2025), but two studies were conducted on sheep (Wu H. et al., 2023; Liu et al., 2020) and one on rabbits (Fan et al., 2020). Defects were most commonly rat calvarial critical-sized defects (Wu P. et al., 2023; Zhao et al., 2024; Zheng et al., 2023; Joo et al., 2024; Wang et al., 2022; Yong et al., 2025), although some studies evaluated load-bearing skeletal sites, including segmental long-bone defects (Wu H. et al., 2023; Liu et al., 2020; Li et al., 2025; Fan et al., 2020). Follow-up periods varied substantially across studies, ranging from 4 weeks up to 12 months post-implantation. Sample sizes ranged from 4 to 40 animals. Bone regeneration was assessed in all the studies via micro-CT analysis and histological evaluation.

**TABLE 1:** Risk of bias was assessed using SYRCLE’s risk of bias tool for animal studies.

Study	Sequence generation	Baseline characteristics	Allocation concealment	Random housing	Blinding (Caregivers/Investigators)	Random outcome assessment	Blinding (outcome assessor)	Incomplete outcome data	Selective outcome reporting	Other bias	Overall judgment
Wu et al. (2023a)	Unclear	Unclear	Unclear	Unclear	Unclear	Unclear	Unclear	Low	Low	Unclear	UNCLEAR: mainly due to insufficient reporting of randomization procedures, allocation concealment, housing conditions, and blinding methods, despite generally complete outcome reporting and absence of evident attrition bias
Wu et al. (2023b)	Unclear	Low	Unclear	Unclear	Unclear	Unclear	Unclear	Low	Unclear	Unclear	UNCLEAR: insufficient details were provided regarding sequence generation, allocation concealment, random housing, and blinding of caregivers and outcome assessors. Incomplete outcome data were judged at low risk, as no attrition or exclusions were reported
Zhao et al. (2024)	Unclear	Low	Unclear	Unclear	Unclear	Unclear	Unclear	Low	Unclear	Unclear	UNCLEAR: key methodological details such as sequence generation, allocation concealment, housing conditions, and blinding procedures were not reported. Incomplete outcome data were judged at low risk, as no attrition or exclusions were described
Zheng et al. (2023)	Unclear	Low	Unclear	Unclear	Unclear	Unclear	Unclear	Unclear	Unclear	High	UNCLEAR: insufficient methodological details were reported regarding sequence generation, allocation concealment, housing conditions, and blinding. A high risk of other bias was identified due to potential unit-of-analysis bias, since bilateral calvarial defects may have been analyzed as independent samples
Joo et al. (2024)	Unclear	Low	Unclear	Unclear	Unclear	Unclear	Unclear	Low	Unclear	Low	UNCLEAR: insufficient methodological details were provided regarding sequence generation, allocation concealment, housing conditions, and blinding procedures. No major additional sources of bias were identified
Liu et al. (2020)	Unclear	Unclear	Unclear	Unclear	Unclear	Unclear	Unclear	Unclear	Unclear	Low	UNCLEAR: No information was provided regarding sequence generation, allocation concealment, housing conditions, or blinding procedures. Incomplete outcome data were also judged as unclear, as it was not specified whether all animals were accounted for at each time point. No major additional sources of bias were identified
Li et al. (2025)	Unclear	Low	Unclear	Unclear	Unclear	Unclear	Unclear	Low	Unclear	Low	UNCLEAR: key methodological details such as sequence generation, allocation concealment, housing conditions, and blinding procedures were not described. No major additional sources of bias were identified
Fan et al. (2020)	Unclear	Low	Unclear	Unclear	Unclear	Unclear	Unclear	Low	Unclear	Low	UNCLEAR: no details were provided regarding sequence generation, allocation concealment, housing conditions, or blinding procedures. Incomplete outcome data were judged at low risk, as no attrition or exclusions were reported
Wang et al. (2022)	Unclear	Low	Unclear	Unclear	Unclear	Unclear	Unclear	Low	Unclear	High	UNCLEAR: no details were provided regarding sequence generation, allocation concealment, or blinding procedures. Importantly, two defects were created per animal, raising concerns about pseudoreplication and non-independence of observations if each defect was analyzed as an independent sample
Yong et al. (2025)	Unclear	Low	Unclear	Unclear	Unclear	Unclear	Unclear	Low	Unclear	High	UNCLEAR: key methodological details such as sequence generation, allocation concealment, and blinding procedures were not described. Furthermore, the use of multiple defects per animal raises concerns regarding non-independence of observations and potential pseudoreplication

### Scaffold composition

All included studies investigated piezoelectric scaffolds capable of generating electrical signals by responding to mechanical stimulation, with the aim of enhancing osteogenesis. The scaffold materials were primarily based on piezoelectric polymers or ceramic-polymer composites: these included poly (vinylidene fluoride) (PVDF) (Li et al., 2025), poly (vinylidene fluoride–trifluoroethylene) (PVDF–TrFE) (Joo et al., 2024), polycaprolactone (PCL) (Wang et al., 2022), barium titanate (BaTiO_3_) (Wu H. et al., 2023; Wu P. et al., 2023; Zheng et al., 2023; Liu et al., 2020; Fan et al., 2020; Yong et al., 2025), or hydroxyapatite (Wu P. et al., 2023; Zhao et al., 2024; Joo et al., 2024). Several studies incorporated piezoelectric ceramic nanoparticles, most commonly BaTiO_3_ (Zheng et al., 2023; Liu et al., 2020; Fan et al., 2020; Yong et al., 2025), into polymer matrices or metallic scaffolds to enable mechanically induced electrical stimulation. Other studies incorporated bioactive ceramics such as hydroxyapatite into polymer matrices or hydrogel systems (Wu P. et al., 2023; Zhao et al., 2024; Joo et al., 2024). In one study, shape-memory functionality was also introduced to facilitate scaffold conformability and defect filling (Li et al., 2025). Scaffolds structural and mechanical properties are summarized in Table 2.

**TABLE 2:** Summary of preclinical studies investigating piezoelectric and smart scaffolds for bone and osteochondral regeneration, including scaffold composition, fabrication methods, functional properties, and biological outcomes.

Author	Model	Sample size	Defect location and size	Scaffold composition	Type of smart functionality	Additive manufacturing process	Structural and mechanical properties	Surface modifications	Exogenous bioactive agents	Follow-up	Outcome measure	Summary of results
Wu et al. (2023a)	Rat + sheep	12 rats; 18 sheep	Cervical vertebral defect (C4 corpectomy, sheep); porous scaffold for subcutaneous implantation (10 mm diameter × 3 mm height, rat)	3D-printed porous Ti6Al4V scaffold coated with BaTiO_3_ and electrically polarized [BT/Ti (poled)]	Piezoelectric stimulation through BaTiO_3_ coating converting mechanical loading/LIPUS into electrical signals	Electron Beam Melting (EBM)	Pore size ∼600 μm; porosity 82%; compressive modulus maintained after coating/polarization; d33 up to 3.2 pC/N; improved wettability	Hydrothermal BaTiO_3_ coating + corona polarization	None	12 months	Micro-CT, histology (H&E, VG), immunofluorescence, macrophage polarization, ALP/ARS staining, biomechanical and metabolic analyses	BT/Ti (poled) scaffolds promoted M2 macrophage polarization, enhanced osteogenesis and osseointegration, increased BV/TV (∼34% at 12 months vs ∼24% in controls), improved trabecular bone formation, reduced inflammation and fibrous tissue response, and activated oxidative phosphorylation pathways supporting bone regeneration
Wu et al. (2023b)	Rat	36	critical-sized calvarial (cranial) bone defect	Chitosan/gelatin hydrogel (CG) doped with PDA-modified hydroxyapatite (PHA) and PDA-modified BaTiO_3_ nanoparticles (PBT); best-performing group: CG/PHA/5%PBT	piezoelectric	None reported; hydrogel prepared by mixing/crosslinking and freeze-drying for characterization	Porous scaffold; uniform distribution of Ca, P, Ba, Ti; roughness increased with nanoparticle loading while porosity remained unchanged; storage modulus: 342.8 Pa (CG), 408.4 Pa (CG/PHA), 493.1 Pa (CG/PHA/5%PBT), 532.2 Pa (CG/PHA/10%PBT); output voltage approximately 0.2 V, 0.25 V, 0.6 V, 0.8 V respectively; tetragonal BaTiO_3_ phase confirmed	Polydopamine (PDA) coating/modification	None	2, 4 and 8 weeks	*In vivo* immunofluorescence/IHC for iNOS, CD206, TNF-α, IL-10, VEGF, CD31, BMP-2, Runx2; micro-CT (BV/TV, BMD); histology (H&E, Masson’s trichrome, Goldner’s trichrome); IHC for Col-1, Runx2, OPN, CD31, CD34	CG/PHA/5%PBT showed the best performance. It promoted M2 macrophage polarization, reduced inflammatory markers, and increased pro-healing cytokines. *In vivo*, it significantly improved vascularization and osteogenesis. At 8 weeks, BV/TV was 35.4% ± 4.1%, higher than CG/PHA (24.3% ± 5.2%) and CG (15.8% ± 3.7%), with higher BMD and more mature bone formation histologically. The scaffold showed abundant mineralized matrix, lamellar bone formation, and strong osteogenic/angiogenic marker expression
Zheng et al. (2023)	Rat (Sprague-Dawley)	8	Critical-sized calvarial bone defect (5 mm diameter)	Scaffold bilayer: B-S layer (chitosan + hydroxyapatite) + P-S layer (gelatin methacryloyl + alginate + piezoelectric whitlockite (PWH))	piezoelectric	Piezoelectric + ultrasound-activated (LIPUS)	3D porous bilayer structure; pore size: 100–350 μm in P-S and 100–200 μm in B-S; compressive strength of Bio-S: 0.77 MPa; compressive modulus: 1.02 MPa; hydrogel piezoelectric coefficient: ∼9.41 pC/N; stable mechano-electric conversion; degradation ∼46% at 35 days	HAp + PWH (Mg^2+^ release, piezoelectricity)	None	8 weeks	Micro-CT (BV/TV, BMD); histology (H&E, Masson’s trichrome); immunofluorescence (CD31, CGRP); RT-qPCR (COL I, Runx2, VEGF, TUBB3); ALP and ARS staining; Western blot; RNA-seq	Bio-S + LIPUS showed the best regenerative performance, with nearly complete defect repair and integration with native bone. Quantitatively, it achieved the highest BV/TV (35.7%) and BMD (0.45 g/cm^3^). It significantly enhanced osteogenesis, angiogenesis, and neurogenesis compared with control groups. Gene and protein expression analyses showed marked upregulation of osteogenic, angiogenic, and neurogenic markers. The proposed mechanism involved activation of PI3K-AKT, MAPK, HIF-1, ATP-related, and Ca^2+^ signaling pathways, with a synergistic effect of piezoelectric stimulation and Mg^2+^ release
Zheng et al. (2023)	Rat (Sprague-Dawley)	40	Critical-sized calvarial bone defect (5 mm diameter) (bilateral defects)	Composite cryogel scaffold (Gel-PD-CMBT): gelatin (Gel) matrix + conductive PEDOT:PSS + piezoelectric Ca/Mn-doped BaTiO_3_ nanofibers (CMBT)	Combined piezoelectric and conductive electroactivity	No additive manufacturing; scaffold fabricated via sol–gel electrospinning (nanofibers), cryogelation, chemical crosslinking, and freeze-drying	Interconnected porous structure (≈88–106 μm pores); porosity ∼58–73%; conductivity up to 0.59 S/cm; compressive modulus increased with composite (highest in Gel-PD-CMBT); good cyclic mechanical stability and shape recovery	homogeneous dispersion of conductive and piezoelectric components	None	4 and 8 weeks	Micro-CT (BV/TV, BMD); histology (H&E, Masson’s trichrome); CCK-8; ALP/ARS staining; RT-qPCR (ALP, OPN, COL-I, OCN); electrical characterization (CV, EIS)	The polarized Gel-PD-CMBT scaffold showed the best performance, with near-complete defect filling at 8 weeks and the highest BV/TV (∼53%) and BMD (∼0.53 g/cm^3^). It significantly enhanced osteogenesis compared to controls. The effect is attributed to the synergistic combination of conductivity and piezoelectricity, which reconstructs the local electroactive microenvironment, promoting cell differentiation and bone formation
Joo et al. (2024)	Mouse (C57Bl/6N)	16	Critical-sized calvarial bone defect (5 mm diameter)	Composite piezoelectric scaffold (HAp/P(VDF-TrFE)): ferroelectric polymer P(VDF-TrFE) + hydroxyapatite (HAp) nanoparticles (1–12 vol%)	Piezoelectric + topographical + paracrine bioactivity	Solvent casting + annealing + corona poling (no 3D printing)	Thin flexible membranes (∼16–18 μm); high β-phase crystallinity; increased piezoelectric coefficient after poling; improved mechanical strength (HAp scaffold > P(VDF-TrFE)); enhanced surface roughness (Ra ∼15.6 nm)	Intrinsic topographical roughness from HAp nanoparticle dispersion	None	2, 4, and 6 weeks	Micro-CT (bone volume/area); histology (H&E, Masson); cell proliferation; ALP/ARS staining; growth factor array; PFM/KPFM	The HAp/P(VDF-TrFE) scaffold showed significantly enhanced bone regeneration compared to controls. This effect is driven by a triple synergistic mechanism: (i) increased piezoelectricity → Ca^2+^ attraction and electrical stimulation; (ii) higher surface roughness → improved cell adhesion and morphology; (iii) upregulation of osteogenic growth factors (e.g., BMP-4, BMP-7, TGF-β). Nearly complete defect healing and superior bone volume were observed at 6 weeks
Liu et al. (2020)	Sheep	18	Segmental femoral defect, 3 cm length, 0.5 cm diameter	Porous Ti6Al4V scaffold coated with BaTiO_3_ (BTi) vs uncoated Ti (pTi)	Piezoelectric (BaTiO_3_) + ultrasound-activated (LIPUS)	Electron Beam Melting (EBM) 3D printing of Ti6Al4V scaffold	High porosity (∼72–74%); compressive strength ∼63–65 MPa (similar to cortical bone); interconnected porous architecture; suitable load-bearing properties	BaTiO_3_ coating (hydrothermal + corona poling)	None	4 and 8 months	ALP activity; gene expression (Runx2, Col-1, OPN); micro-CT (BV/TV); histology (Van Gieson); X-ray	BaTiO_3_-coated scaffolds (BTi) showed significantly enhanced osteogenesis vs Ti alone: ↑ ALP activity, ↑ osteogenic gene expression, ↑ mineralization *in vitro*; *in vivo* → greater bone ingrowth, higher BV/TV, improved osseointegration at 4 and 8 months. LIPUS-activated piezoelectric effect promoted Ca^2+^ signaling and osteoblast differentiation, leading to superior repair of large segmental defects
Li et al. (2025)	Rat	Not clearly reported	Femoral defect, 3 mm diameter × 2 mm depth	D-printed composite scaffold (PVDF + SMPU) combining piezoelectric polymer (PVDF) and shape-memory polyurethane (SMPU)	Piezoelectric + shape-memory + self-powered (mechanical stimulation-induced electrical output); also immunomodulatory	3D printing + electrospinning (PVDF nanofibers + SMPU printable scaffold)	3D-printed porous SMPU/PVDF composite scaffold; NaCl porogen (60–80 µm); compressive modulus 2.4 ± 0.4 MPa; tensile modulus of SMPU ≈88 MPa high elasticity (strain up to 1,164%)	None	None	8 weeks	Cell viability (BMSCs, RAW264.7); macrophage polarization (M1/M2); ALP/ARS; gene expression (Runx2, OCN, OPN, COL1); micro-CT (BV/TV, BMD); histology	Scaffold showed excellent cytocompatibility and strong immunomodulatory effect, promoting M2 macrophage polarization and reducing inflammation. Under mechanical stimulation, it generated electrical signals enhancing osteogenic differentiation. *In vivo*, it achieved significantly improved bone regeneration, with higher BV/TV and BMD vs controls. Shape-memory allowed minimally invasive implantation and defect filling, while self-powered piezoelectricity enabled continuous stimulation during rehabilitation
Fan et al. (2020)	Rabbit	96	Femoral condyle defect (6 mm diameter × 10 mm length)	Porous Ti6Al4V scaffold coated with BaTiO_3_ (piezoelectric ceramic)	Piezoelectric (externally activated via LIPUS)	3D printed porous titanium scaffold (CAD + additive manufacturing)	Porosity ∼70%; pore size ∼500 μm; compressive strength ∼60 MPa; elastic modulus ∼3–4 MPa	Hydrothermal BaTiO_3_ ceramic coating	None	6 and 12 weeks	Cell proliferation (BMSCs), ALP, gene expression (Runx2, COL1, OPN), micro-CT (BV/TV), histology, osseointegration	BaTiO_3_ coating significantly improved osteogenesis and osseointegration, especially when combined with LIPUS stimulation. The piezoelectric effect enhanced cell adhesion, proliferation, and differentiation. *In vivo*, the BaTiO_3_/Ti + LIPUS group showed the highest bone formation, with increased BV/TV and more mature bone compared to controls
Wang et al. (2022)	Rat	16	Critical-sized calvarial bone defect (5 mm diameter)	3D printed PCL scaffold + β-TCP + piezoelectric whitlockite (PWH, Mg^2+^-doped Ca phosphate)	Self-powered piezoelectric + sustained Mg^2+^ release (bioactive + electroactive)	3D printing (extrusion-based composite scaffold)	3D-printed PCL/PWH composite scaffold (30 wt% piezoelectric whitlockite nanoparticles, ≈85 nm) Porous architecture Compressive stress ≈15 MPa at ∼35% strain	None	None	4 and 8 weeks	Cell viability (BMSCs), ALP, osteogenic gene expression (Runx2, COL1, OCN), angiogenesis (VEGF), neurogenesis (TUBB3, NEFL), micro-CT (BV/TV, BMD), histology	PWH scaffold significantly enhanced osteogenesis, angiogenesis, and neurogenesis compared to controls. The combination of piezoelectricity + Mg^2+^ release showed a synergistic effect, increasing bone formation (higher BV/TV and BMD) and promoting vascularization and innervation
Yong et al. (2025)	Rat	40	Mandibular defect (critical-size: 5 mm)	Collagen decalcified bone matrix gel barium titanate (COL/DBM/BT) scaffold	Piezoelectric (BT-based) + ultrasound-activated (external trigger)	Freeze-drying (porous scaffold fabrication)	ECM-mimicking porous scaffold with interconnected pores enabling bone and vascular ingrowth Adequate mechanical stability for bone regeneration (no detailed quantitative values reported)	No coating; BT nanoparticles embedded in matrix	None	6 and 12 weeks	ell proliferation (BMSCs, HUVECs), ALP, osteogenic markers (Runx2, OPN), angiogenesis assays, antibacterial tests, micro-CT (BV/TV), histology	The CDB + ultrasound group showed the best performance, with significantly enhanced osteogenesis, angiogenesis, and antibacterial activity. Piezoelectric stimulation amplified DBM bioactivity, leading to improved bone regeneration and faster defect closure

### Scaffold fabrication

Additive manufacturing techniques were employed in multiple studies to produce scaffolds with precisely controlled architecture. In particular, porous scaffold fabrication techniques such as electron beam melting (EBM) (Wu H. et al., 2023; Liu et al., 2020) and selective laser melting (SLM) (Fan et al., 2020) were used. 3D-printing methods were employed in other studies, such as fused deposition modeling (FDM) (Li et al., 2025), extrusion-based printing (melt extrusion) (Wang et al., 2022) and electrohydrodynamic jet (EHD) printing/near-field electrospinning–based 3D printing (Joo et al., 2024). Surface modifications were reported in several studies and included ceramic nanoparticle incorporation, piezoelectric polarization, and composite scaffold engineering. Exogenous growth factors or pharmacological agents were never incorporated. All the scaffolds relied on intrinsic electroactive properties to stimulate bone regeneration.

### Imaging outcomes

Micro-computed tomography (micro-CT) analysis consistently demonstrated increased bone volume fraction (BV/TV) and bone mineral density (BMD) in the smart scaffold groups. Micro-CT evaluation consistently demonstrated higher bone volume fraction (BV/TV) and bone mineral density (BMD) in the experimental groups (Wu H. et al., 2023; Wu P. et al., 2023; Zhao et al., 2024; Zheng et al., 2023; Joo et al., 2024; Liu et al., 2020; Li et al., 2025; Fan et al., 2020; Wang et al., 2022; Yong et al., 2025). In rodent calvarial defect models, several piezoelectric scaffolds achieved BV/TV values of approximately 35%–36% at 8 weeks, indicating substantial bone formation within the defect (Wu P. et al., 2023; Zhao et al., 2024; Zheng et al., 2023; Joo et al., 2024; Wang et al., 2022; Yong et al., 2025). The CG/PHA/5%PBT piezoelectric hydrogel reported by Wu et al. achieved BV/TV values of approximately 35.4% at 8 weeks (Wu P. et al., 2023), together with significantly increased BMD compared with CG/PHA and CG scaffolds. Similarly, Bio-S scaffolds combined with low-intensity pulsed ultrasound (LIPUS) achieved BV/TV values of 35.7% and BMD of 0.45 g/cm^3^ (Zhao et al., 2024). Other piezoelectric scaffold systems also demonstrated significant improvements in bone regeneration. Polarized Gel-PD-CMBT scaffolds significantly increased BV/TV and BMD at both 4 and 8 weeks (Zheng et al., 2023), while shape-memory piezoelectric scaffolds significantly improved BV/TV and BMD at 4 and 8 weeks (Li et al., 2025), with micro-CT reconstructions showing greater new bone formation and improved defect bridging. Likewise, PWH scaffolds significantly increased BV/TV and BMD at 4 and 8 weeks compared with PCL, β-TCP, and WH controls, with markedly improved defect closure (Wang et al., 2022). In another study, BaTiO_3_-coated titanium scaffolds combined with LIPUS demonstrated the highest BV/TV and BMD at 6 and 12 weeks, together with improved defect bridging and stronger bone–implant bonding strength (Fan et al., 2020). Similarly, barium titanate-coated titanium scaffolds exhibited significantly higher BV/TV compared with controls at 4 and 8 months (Yong et al., 2025).

### Histological findings

Histological analyses consistently confirmed the imaging findings across the included studies. Staining techniques included hematoxylin–eosin (H&E), Masson’s trichrome, Van Gieson, and Goldner’s trichrome. These demonstrated dense trabecular bone formation, abundant mineralized matrix deposition, and improved structural organization of the regenerated tissue. In the CG/PHA/5%PBT hydrogel study, histological staining showed well-organized lamellar bone formation with bone lacunae and central canal-like structures, indicating more mature bone regeneration compared with control scaffolds (Wu P. et al., 2023). Similarly, Bio-S + LIPUS scaffolds achieved near-complete defect bridging, with extensive mature bone and collagen deposition (Zhao et al., 2024). Polarized Gel-PD-CMBT scaffolds produced dense, well-organized new bone with abundant collagen deposition and minimal fibrous tissue (Zheng et al., 2023), while HAp/P(VDF-TrFE) scaffolds demonstrated greater mineralized matrix deposition and more complete defect bridging compared with control groups (Joo et al., 2024). In addition to increased bone formation, several studies reported improved scaffold–bone integration and reduced fibrous tissue formation. For instance, BaTiO_3_-coated titanium scaffolds combined with LIPUS resulted in dense trabecular bone formation at the bone–implant interface and reduced fibrous tissue interposition (Fan et al., 2020). In a large-animal vertebral defect model, BT/Ti (poled) scaffolds demonstrated significantly greater bone regeneration compared with conventional titanium scaffolds (Wu H. et al., 2023). At 12 months, BV/TV reached approximately 34% in BT/Ti (poled) scaffolds compared with approximately 24% in titanium controls, together with increased trabecular thickness and tight integration with the surrounding host bone (Wu H. et al., 2023). Histological evaluation confirmed abundant dense trabecular bone within and around the scaffolds, accompanied by reduced inflammatory reactions and fewer foreign body giant cells.

## Discussion

To our knowledge, this is the first systematic review of the *in vivo* evidence on piezoelectric scaffolds for bone repair. This review analyzed the current preclinical evidence regarding smart piezoelectric bone scaffolds for bone regeneration. Across the included studies, electroactive scaffolds consistently demonstrated improved bone regeneration compared with conventional non-smart scaffolds, as evidenced by micro-CT and histological analysis. These benefits were observed across polymer-based, ceramic-based, and composite scaffolds, and were associated with increased bone volume fraction and bone mineral density, and improved structural organization of the regenerated tissue.

These findings support the hypothesis that scaffolds capable of generating or transmitting electrical signals could better replicate the dynamic microenvironment of native bone tissue and enhance the regenerative process. Conventional scaffolds can provide structural support and osteoconductive surfaces, but they generally remain passive biomaterials and do not reproduce the endogenous electromechanical signals involved in bone remodeling and repair (Huang et al., 2022; Liu et al., 2026). In critical-sized defects, conventional scaffolds may fail to provide adequate biological stimulation and mechanical support, resulting in inferior repair and possible progression to nonunion (Huang et al., 2022; Liu et al., 2026; Leng et al., 2019). In this scenario, piezoelectric materials could play a role in promoting osteogenesis and tissue integration. These can be organic piezoelectric materials, such as polymers or natural biomacromolecules, or inorganic piezoelectric biomaterials, mainly ceramics or crystals (Dong et al., 2026; Dong et al., 2021). The included studies investigated scaffolds incorporating piezoelectric ceramics such as BaTiO_3_ (Wu H. et al., 2023; Wu P. et al., 2023; Zheng et al., 2023; Liu et al., 2020; Fan et al., 2020; Yong et al., 2025) or piezoelectric polymers such as PVDF and PVDF-TrFE (Joo et al., 2024; Li et al., 2025). These materials can convert mechanical forces into electrical stimulation, mimicking the endogenous electromechanical signals naturally present in bone tissue during loading. Natural bone tissue generates endogenous electrical signals during mechanical loading through both piezoelectricity and streaming potentials, which are essential for bone remodeling and self-repair (Vasquez-Sancho et al., 2018; Ren et al., 2015; Khare et al., 2020). Collagen piezoelectricity has traditionally been considered the primary source (Vasquez-Sancho et al., 2018). Recent evidence demonstrates that hydroxyapatite exhibits flexoelectricity, which is voltage generation in response to strain gradients (Vasquez-Sancho et al., 2018). This may be the main source of bending-induced polarization in cortical bone (Vasquez-Sancho et al., 2018). Instead, streaming potentials are electrokinetic phenomena generated when interstitial fluid flows through the lacunocanalicular network of bone in response to mechanical loading (Ren et al., 2015). These electromechanical signals serve as the biological basis for biomimetic piezoelectric scaffolds: these materials convert physiological mechanical stimuli into localized electrical signals, thereby reproducing endogenous electromechanical cues (Khare et al., 2020). Upon deformation, piezoelectric scaffolds generate surface charges that promote calcium-mediated signaling and activation of osteogenic pathways such as MAPK and Wnt/β-catenin (Dong et al., 2026; Dong et al., 2021). This electromechanical coupling creates a self-powered mechanotransductive feedback loop that supports mineralization and improves scaffold–bone integration (Farjaminejad and Dingle, 2025; Leng et al., 2019). Similarly, in piezoelectric ceramics such as BaTiO_3_, mechanical deformation alters the relative positions of ions within a non-centrosymmetric crystal lattice, producing electrical polarization and surface charge generation (Huang et al., 2025). In piezoelectric polymers such as PVDF and PVDF–TrFE, the electromechanical response is primarily associated with the alignment of polar molecular dipoles, particularly in the electroactive β-phase, enabling mechanical deformation to generate measurable electrical potentials (Ahbab et al., 2025). These materials can therefore convert physiological forces into localized electrical cues that stimulate osteogenic cell activity and matrix mineralization (Huang et al., 2025; Ahbab et al., 2025). Consistent with these mechanisms, the studies included in this review reported significantly higher bone volume fraction (BV/TV) and bone mineral density (BMD) in piezoelectric scaffold groups compared with controls.

The structural design of scaffolds also emerged as an important factor influencing regenerative outcomes. Included scaffolds presented highly interconnected porous architectures with pore sizes ranging from approximately 100–800 μm. The literature strongly supports the 100–800 μm pore size range for bone tissue engineering scaffolds, with extensive evidence demonstrating that this range optimizes cell infiltration, vascularization, and nutrient diffusion through highly interconnected porous architectures (Karageorgiou and Kaplan, 2005). Smaller pores (100–300 μm) favor initial cell attachment and early tissue formation (Karageorgiou and Kaplan, 2005), whereas larger pores (>300 μm) promote vascularized bone formation and long-term remodeling (Luo et al., 2021). Importantly, interconnectivity and permeability are critical, as they regulate nutrient transport, waste removal, and mechanotransduction (Mukasheva et al., 2024).

Mechanical properties also varied considerably among scaffold types. Metallic scaffolds based on porous Ti6Al4V alloys combined with barium titanate (BaTiO_3_) exhibited compressive moduli in the range of hundreds of megapascals to several gigapascals (Liu et al., 2020; Fan et al., 2020; Yong et al., 2025). This high strength and fatigue resistance allow them to maintain defect space and support physiological loads (Ntakos et al., 2025; Yavari et al., 2013). However, these materials are non-degradable and may cause stress shielding due to stiffness mismatch with native bone (Hériveaux et al., 2022). In contrast, polymeric and hydrogel-based scaffolds (Wu H. et al., 2023; Wu P. et al., 2023; Zhao et al., 2024; Zheng et al., 2023; Joo et al., 2024; Li et al., 2025; Wang et al., 2022) typically exhibit lower mechanical stiffness, which limits their use in load-bearing sites (Gibbs et al., 2016; Bakhtiari et al., 2023). Scaffold requirements differ by anatomical site: load-bearing bones need high strength and fatigue resistance, whereas non-load-bearing craniofacial/skull sites require more shape adaptability and softer biomechanical matching (Dong et al., 2026; Dong et al., 2021). However, many of the included studies relied on rat calvarial defect models (Wu P. et al., 2023; Zhao et al., 2024; Zheng et al., 2023; Joo et al., 2024; Wang et al., 2022; Yong et al., 2025), which are subjected to only limited cyclic mechanical loading. This may restrict activation of the piezoelectric effect and therefore limit the extent to which these findings can be extrapolated to load-bearing skeletal sites.

Nevertheless, they offer several advantages, including greater flexibility and a biomimetic extracellular matrix-like environment that promotes cell adhesion and proliferation (Bakhtiari et al., 2023; Fan et al., 2023; Farjaminejad et al., 2024). Composite systems combining polymers, ceramics, and bioactive nanoparticles attempted to balance these characteristics by providing adequate mechanical support while maintaining bioactivity and electroactive functionality (Ansari et al., 2024).

Thus, scaffold architecture directly influences biological and mechanical properties of the scaffold.

Additive manufacturing technologies, including electron beam melting (EBM) (Wu H. et al., 2023; Liu et al., 2020), selective laser melting (SLM) (Fan et al., 2020), fused deposition modeling (FDM) (Li et al., 2025), and extrusion-based printing (Wang et al., 2022), were widely used to fabricate scaffolds with controlled architectures and reproducible mechanical properties. These technologies allow precise control of porosity and scaffold geometry, which are essential for optimizing both mechanical performance and biological integration. In particular, additive manufacturing can control the spatial distribution of the piezoelectric phase, potentially ensuring a more homogeneous functional response throughout the scaffold (Gon et al., 2025). Moreover, by regulating filament or fiber orientation, additive manufacturing may influence scaffold anisotropy and the directionality of force transmission, which in turn can affect the magnitude and distribution of the electrical cues generated under mechanical loading (Chi et al., 2022). In multilayer or composite systems, the layer-by-layer organization enabled by additive manufacturing may further modulate how stresses are transferred across the scaffold and how different material phases interact during loading (Gon et al., 2025). Moreover, piezoelectric coatings on conventional scaffolds may suffer from interfacial mismatch and limited long-term functional stability, whereas additive manufacturing of piezoelectric architectures may offer better structural integration (Chen A. et al., 2026). At the same time, several studies employed conventional fabrication methods such as freeze-drying or electrospinning to produce porous scaffolds, particularly for hydrogel-based systems (Wu P. et al., 2023; Zheng et al., 2023; Yong et al., 2025). Indeed, heterogeneity of fabrication methods used across the included studies was observed. Importantly, additive manufacturing primarily determines the structural design of the scaffold rather than its functional activation. In other words, it can optimize porosity and spatial distribution of the piezoelectric phase, but it does not by itself ensure generation of biologically relevant electrical cues. Recent work on autonomous piezoelectric implants suggested that the clinical value of piezoelectric biomaterials may depend not only on their osteogenic potential, but also on how effectively they can be activated under physiological conditions (Chen A. et al., 2026). In several studies (Zhao et al., 2024; Liu et al., 2020; Fan et al., 2020), this functional component was enhanced through external mechanical stimulation, most commonly low-intensity pulsed ultrasound (LIPUS), which activated the piezoelectric scaffold and promoted localized electrical signal generation. These localized electrical signals—particularly in BaTiO_3_-containing systems (Wu H. et al., 2023; Wu P. et al., 2023; Zheng et al., 2023; Liu et al., 2020; Fan et al., 2020; Yong et al., 2025)— lead to enhanced osteogenesis, improved bone–implant integration, and greater mechanical stability of regenerated bone. Collectively, this evidence indicates that external stimulation may amplify the regenerative potential of piezoelectric materials, although the clinical feasibility of such approaches requires further investigation. Chen et al. observed that in occlusion-activated dental implants endogenous biomechanical loading may provide a clinically attractive alternative to external stimulation strategies such as ultrasound or magnetic fields (Chen A. et al., 2026). This could improve practicality and long-term integration, although its effectiveness may remain dependent on the local mechanical environment of the implantation site.

Histologically, nonunion is characterized by persistence of fibrous and/or fibrocartilaginous tissue, incomplete or disorganized woven bone formation, leading to poor bridging across the fracture gap and failure to remodel into mature lamellar bone (Panteli et al., 2015; Hossen et al., 2025). Instead, histological analyses across the included studies consistently supported the imaging findings and provided additional insight into the quality of the regenerated tissue. Staining techniques repeatedly demonstrated denser trabecular bone formation, more extensive collagen-rich matrix deposition, and improved scaffold–bone integration in piezoelectric scaffold groups (Wu H. et al., 2023; Wu P. et al., 2023; Zhao et al., 2024; Zheng et al., 2023; Joo et al., 2024; Fan et al., 2020). The regenerated tissue also showed more advanced structural organization, with better defect bridging and evidence of more mature bone architecture, including lamellar and bone lacunae arrangement, or central canal-like structures (Wu H. et al., 2023; Wu P. et al., 2023; Zhao et al., 2024; Zheng et al., 2023; Joo et al., 2024; Fan et al., 2020). Piezoelectric systems were also more frequently associated with reduced fibrous tissue interposition, indicating a more favorable transition from early repair tissue to mineralized bone (Wu H. et al., 2023; Wu P. et al., 2023; Zhao et al., 2024; Zheng et al., 2023; Joo et al., 2024; Fan et al., 2020). In normal bone healing, early granulation and fibrous repair tissue are progressively replaced by woven bone and subsequently by mineralized bone (Einhorn and Gerstenfeld, 2015). Accordingly, the persistence of a fibrous layer between the scaffold and host bone may reflect incomplete transition to direct bone apposition and delayed osseointegration, meaning a less mechanically continuous repair (Einhorn and Gerstenfeld, 2015; Sheen and Garla, 2023). By contrast, the reduced fibrous tissue interposition observed in several piezoelectric scaffold studies may suggest more direct bone–scaffold contact and a more advanced stage of tissue maturation. This may be secondary to the ability of piezoelectric materials to generate localized electrical cues that stimulate osteogenic cell activity and enhance matrix deposition, thereby favoring earlier differentiation of reparative cells toward bone-forming phenotypes.

An important issue in interpreting the present findings is that many included studies used rat calvarial defect models (Wu P. et al., 2023; Zhao et al., 2024; Zheng et al., 2023; Joo et al., 2024; Wang et al., 2022; Yong et al., 2025), which are only minimally exposed to the cyclic mechanical loads that normally activate piezoelectric materials *in vivo*. This is a relevant limitation, because the biological rationale of piezoelectric scaffolds relies on the conversion of mechanical deformation into localized electrical cues (Chen A. et al., 2026). In non-load-bearing models, such activation may be inconsistent or dependent on indirect sources of deformation, such as soft tissue movement or local pressure transmission, which are unlikely to reproduce the magnitude and pattern of loading experienced in long bones or vertebral sites. As a result, the regenerative effects observed in calvarial models may not fully reflect the performance of piezoelectric scaffolds under physiologically relevant loading conditions. Therefore, the predominance of non-load-bearing models in the current literature should be considered an important translational limitation, and future research should place greater emphasis on load-bearing defects and mechanically relevant *in vivo* conditions. Notably, some studies attempted to overcome this limitation through external activation via low-intensity pulsed ultrasound (LIPUS) (Wu H. et al., 2023; Zhao et al., 2024; Liu et al., 2020; Fan et al., 2020), which likely enhanced electromechanical stimulation. However, this further complicates interpretation, because the observed benefit may derive from the interaction between the scaffold and the external stimulus rather than from spontaneous activation alone. From a future perspective, emerging scaffold designs may further enhance the performance of piezoelectric systems by improving their interaction with external stimuli. Ultrasound-responsive lattice architectures may optimize energy transmission and local stress concentration, thereby improving activation of the piezoelectric phase (Chen Z. et al., 2026). Similarly, stimuli-responsive or 4D-printed scaffolds may introduce adaptive structural behavior over time (Wang et al., 2025). Together, these advances point toward a shift from passive constructs to more adaptive systems designed to interact dynamically with the healing environment.

Other several limitations should be acknowledged. First, all included studies were preclinical investigations, primarily conducted in small animal models. Although some studies employed large animal models, including sheep and rabbits, clinical translation remains limited. Second, considerable heterogeneity was observed among studies in terms of scaffold composition, fabrication methods, defect models, and follow-up duration. This variability makes direct comparisons challenging and limits the ability to perform quantitative meta-analysis. Third, sample sizes were generally small and follow-up periods were relatively short, with most studies evaluating outcomes within 4–12 weeks after implantation. This is particularly relevant for scaffold systems based on BaTiO_3_-containing ceramics (Dong et al., 2026) and PVDF/PVDF-TrFE-based polymers (Chen Z. et al., 2026), because these materials are not rapidly resorbed and may persist for prolonged periods after implantation. In the case of slowly degrading polymers, prolonged persistence may help preserve electromechanical functionality and structural support, but may also limit clearance and extend exposure at the implantation site. Similarly, ceramic piezoelectric phases may be beneficial for functional activation yet still require more detailed long-term biocompatibility assessment. The currently available evidence is insufficient to draw conclusions about long-term degradation behavior and potential local or systemic toxicity. Another important point concerns the internal validity of the included studies. Table 1 shows that most studies were rated as “unclear” in critical SYRCLE domains, particularly allocation concealment, random housing, and blinding of outcome assessors. Such methodological shortcomings may lead to exaggerated estimates of scaffold efficacy. For example, inadequate control of group allocation and housing conditions can introduce systematic bias, whereas non-blinded assessment may favor more positive interpretation of regenerative outcomes. Consequently, the beneficial effects observed for piezoelectric scaffolds may represent an overestimation of their true regenerative performance. This limitation should be kept in mind when interpreting the consistency of positive findings across studies. Future research should therefore aim to establish standardized experimental protocols and reporting guidelines to facilitate comparison between studies and accelerate clinical translation.

## Conclusions

Overall, the findings of this systematic review suggest that smart piezoelectric scaffolds represent a promising strategy for enhancing bone regeneration. Across the included preclinical studies, these systems consistently demonstrated superior regenerative performance compared with conventional non-piezoelectric scaffolds. These benefits were observed across different piezoelectric material classes, including polymer-based, ceramic-based, and composite systems, suggesting that electromechanical stimulation may provide a biologically relevant advantage in bone healing. An additional contribution of this review is the identification of scaffold design and fabrication as important determinants of performance. In particular, additive manufacturing appears to play a role by enabling more precise control over scaffold architecture. Nevertheless, the current evidence base remains limited by substantial heterogeneity, as well as by the exclusively preclinical nature of the available studies. Future research should therefore focus on more standardized and clinically relevant experimental designs, direct comparison among piezoelectric material platforms, and validation in large-animal load-bearing models. Further clarification of mechanistic pathways and practical activation strategies will also be important to support translation.
